# Development of a DNA-Based Lateral Flow Strip Membrane Assay for Rapid Screening and Genotyping of Six High-Incidence STD Pathogens

**DOI:** 10.3390/bios14050260

**Published:** 2024-05-20

**Authors:** Gunho Choi, Keum-Soo Song, Satish Balasaheb Nimse, Taisun Kim

**Affiliations:** 1 Biometrix Technology, Inc., 2-2 Bio Venture Plaza 56, Chuncheon 24232, Republic of Korea; ghchoi@bmtchip.com (G.C.); hanlimsk@empas.com (K.-S.S.); 2 Institute of Applied Chemistry and Department of Chemistry, Hallym University, Chuncheon 24252, Republic of Korea

**Keywords:** screening, genotyping, sexually transmitted disease, *Chlamydia trachomatis* (CT), *Neisseria gonorrhoeae* (NG), *Trichomonas vaginalis* (TV), *Ureaplasma urealyticum*(UU), *Mycoplasma hominis* (MH), *Mycoplasma genitalium* (MG)

## Abstract

Sexually transmitted diseases (STDs) are a global concern because approximately 1 million new cases emerge daily. Most STDs are curable, but if left untreated, they can cause severe long-term health implications, including infertility and even death. Therefore, a test enabling rapid and accurate screening and genotyping of STD pathogens is highly awaited. Herein, we present the development of the DNA-based 6STD Genotyping 9G Membrane test, a lateral flow strip membrane assay, for the detection and genotyping of six STD pathogens, including *Trichomonas vaginalis*, *Ureaplasma urealyticum*, *Neisseria gonorrhoeae*, *Chlamydia trachomatis*, *Mycoplasma hominis*, and *Mycoplasma genitalium*. Here, we developed a multiplex PCR primer set that allows PCR amplification of genomic materials for these six STD pathogens. We also developed the six ssDNA probes that allow highly efficient detection of the six STD pathogens. The 6STD Genotyping 9G Membrane test lets us obtain the final detection and genotyping results in less than 30 m after PCR at 25 °C. The accuracy of the 6STD Genotyping 9G membrane test in STD genotyping was confirmed by its 100% concordance with the sequencing results of 120 clinical samples. Therefore, the 6STD Genotyping 9G Membrane test emerges as a promising diagnostic tool for precise STD genotyping, facilitating informed decision-making in clinical practice.

## 1. Introduction

Sexually transmitted diseases (STDs) affect millions globally, with an estimated 1 million new cases of curable STDs occurring daily [1,2]. Many of these STDs, when left untreated, can have devastating consequences, leading to long-term health complications, infertility, and even death in some cases [3,4,5,6]. Therefore, treatment of STDs based on early screening and diagnosis is critical in stopping the spread of STDs and avoiding fatal complications [7,8]. Unfortunately, limited access to diagnostic tests obstructs the screening and diagnosis of new STDs in low- and middle-income countries [9]. Therefore, a test that can allow rapid and accurate screening and genotyping of STD pathogens is decisive in the effective management of the associated diseases.

The traditional diagnostic methods, such as cervical cytology, cell culture, immunoassay, and serological testing, are often time-consuming and labor-intensive, leading to delays in STD diagnosis and treatment initiation [10]. The other drawbacks of conventional methods, including low sensitivity and false-negative outcomes, lead to the spread of infection and increased complications in the treatment of disease due to drug resistance [11,12,13]. Therefore, these limitations underscore the necessity for developing a test that can allow rapid genotyping of STD pathogens with high sensitivity and specificity.

Nucleic acid amplification tests (NAAT), employing polymerase chain reaction (PCR), offer notable advantages over conventional methods due to their ability to detect pathogens with exceptional sensitivity and specificity [14,15]. However, interfering materials in clinical specimens have been found to drastically hamper the performance of the PCR-based STD pathogen detection tests [16]. Moreover, the demand for skilled personnel to conduct specimen testing and the prerequisite for advanced laboratory facilities have restricted the utilization of these assays to specialized, high-resource settings. Thus, a test that allows highly specific screening and genotyping of STD pathogens with rapid turnaround time in point-of-care settings is highly awaited [17].

The lateral flow strip membrane (LFSM) assays detect the bacterial and viral genotypes with high sensitivity and specificity [18,19]. Thus, we hypothesize that an LFSM assay can be developed to simultaneously detect multiple nucleic acids associated with STD pathogens in a single test and present itself as a practical alternative to conventional methods such as culture, PCR, or immunoassays. Furthermore, LFSM-based assays offer the advantages of rapidity and simplicity. Therefore, the development of a novel method that can be utilized independently or in conjunction with PCR for the diagnosis and monitoring of suspected STD cases holds promise for mitigating community transmission and controlling the spread of STDs.

Among the various STD pathogens, *Chlamydia trachomatis* (CT), *Neisseria gonorrhoeae* (NG), *Trichomonas vaginalis* (TV), *Ureaplasma urealyticum* (UU), *Mycoplasma hominis* (MH), and *Mycoplasma genitalium* (MG) pose a significant challenge due to their high prevalence in STDs [20,21,22,23,24]. The coinfection of these pathogens is also well-known [25,26]. Therefore, a test that can simultaneously detect STD pathogens is highly desirable [27].

In this context, we present the development of a 6STD Genotyping 9G Membrane test, a multiplex LFSM assay, designed for simultaneous genotyping of six STD pathogens, including CT, NG, TV, UU, MH, and MG. The 6STD Genotyping 9G Membrane test follows a simple experimental protocol, with a 20 m hybridization step, 8 m washing step, and less than a 1 m scanning step, to obtain the final detection and genotyping results in less than 30 m at 25 °C. We evaluated the applicability of the 6STD Genotyping 9G Membrane test for detecting and genotyping STD pathogens in 120 clinical samples. The 100% concordance of the 6STD Genotyping 9G Membrane test with sequencing analysis ensures the high applicability of this test for screening and genotyping of STD pathogens in routine clinical check-ups.

## 2. Materials and Methods

### 2.1. Materials

All reagents were procured from Sigma-Aldrich Chemicals, Seoul, Republic of Korea. High-performance liquid chromatography (HPLC)-grade washing solvents for the substrates were sourced from SK Chemicals, Seoul, Republic of Korea. Ultrapure water (18 M·Ω/cm) was obtained using a Milli-Q purification system (Millipore, Darmstadt, Germany). The PCR premix (AccuPower^®^ PyroHotStart Taq PCR Pre-Mix) and bacterial genomic DNA extraction kits were obtained from Bioneer Corp., Daejeon, Republic of Korea. All standard template DNA, primers, and synthetic oligonucleotide probes designed and used in this study were obtained from Bioneer Corp., Daejeon, Republic of Korea. Oligonucleotides were deposited using a dispenser (Bio-Dot Technologies, Inc., Irvine, CA, USA). Hybridization was conducted at 25 °C without the need for specialized equipment. Fluorescence signal intensities were recorded and analyzed using a BMT Membrane Reader™ (Biometrix Technology Inc., Chuncheon, Republic of Korea).

### 2.2. Clinical Samples

The 120 urine samples, including 60 negative and 60 positive samples comprising ten each for the CT, NG, TV, UU, MH, and MG, were collected from subjects as part of registered protocols approved by the Institutional Review Board (IRB) of U2Bio, Seoul, Republic of Korea. All patients provided written, informed consent (IRB registration number: IRB_2020021).

### 2.3. Nucleic Acid Extraction from the Clinical Specimens and Polymerase Chain Reaction (PCR) Amplification

According to the manufacturer’s instructions, total DNA clinical specimens were extracted from 400 µL of each clinical sample using the ExiPrep™ 96 Genomic DNA Kit (Bioneer, Daejeon, Republic of Korea). The DNA samples were eluted with 100 µL of nuclease-free water and stored at −80 °C until use; 10 µL of this solution was used for the PCR.

The reaction components included 10 μL of a template (Table S1 in Supplementary Materials) solution (or a solution containing extracted DNA), 10 μL of 2X Primer mix designed in this study, and lyophilized PCR premix consisting of 1U of Taq DNA polymerase, 250 μM of dNTP mixture, reaction buffer with 1.5 mM of MgCl_2_, stabilizer, and tracking dye. After vortex mixing and centrifugation, the reaction tube was transferred to the thermocycler SimpliAmp (Thermo Fisher Scientific, Waltham, MA, USA). The PCR amplification contained the following steps: 94 °C for 5 min, followed by 50 cycles at 94 °C for 15 s, 50 °C for 30 s, 72 °C for 10 s, and then 72 °C for 5 min. 20 µL of the Cy5 labeled PCR products were used to detect STD pathogens on the developed 6STD Genotyping 9G Membranes.

### 2.4. Composition of Various Solutions Used

Various solutions were prepared for immobilizing oligonucleotide probes, blocking buffer solution, hybridizing PCR products on the LFSM assay strip, and washing unreacted reagents. The composition of these solutions is as follows. The immobilization solution (pH = 7.4) was obtained by mixing glycerol (15%), butyl amine (50 mM), and NH_4_Cl (600 mM) to the indicated final concentrations. Similarly, the blocking buffer solution (pH = 7.4) was prepared by mixing milk casein (0.5%) in 4 × SSC. The hybridization buffer (pH = 7.4) was obtained by mixing formamide (25%) and Triton X-100 (0.1%) in 6 × SSC. The washing buffer solution (pH = 7.4) was obtained by mixing SDS (0.1%) in 4 × SSC.

### 2.5. Preparation of 9G DNAChips for Selection of Six STD Genotyping Probes

The selection of oligonucleotide probes (Table 1) corresponding to each of the six pathogens studied here was conducted using our previously reported generalized probe selection method for DNA chips [28,29]. The 9G DNAChips were prepared by immobilizing the selected oligonucleotide probes containing nine consecutive guanines on the AMCA glass slides by following the previously reported method [30,31]. These generated 9G DNAChips were utilized to finalize the six probes, each specifically targeting one of the six STD pathogens.

In brief, 20 μL of Cy5-labeled PCR product corresponding to each of the six STD pathogens was initially mixed with 50 μL of hybridization buffer solution. This mixture was then applied to the 9G DNA chips and incubated at 25 °C for 30 m in a commercial incubator. Subsequently, the 9G DNAChips were subjected to rinsing with washing buffer solutions A and B consecutively, each for 2 m at 25 °C, followed by drying in a commercial centrifuge (1000 rpm). Fluorescence signal images obtained using ScanArrayLite (Packard BioChip Technologies, Meriden, CT, USA) were analyzed using Quant Array software to identify the most suitable probes for detecting each of the six STD pathogens. The probe demonstrating highly specific hybridization with the corresponding PCR product of the STD pathogen while exhibiting no non-specific hybridization with PCR products of other STD pathogens was selected as the best probe. Such six probes, each corresponding to one of the six STD pathogens, were then used to fabricate the 6STD Genotyping 9G Membranes.

### 2.6. Typical Method for Preparation of the 6STD Genotyping 9G Membranes

**Scheme 1:**
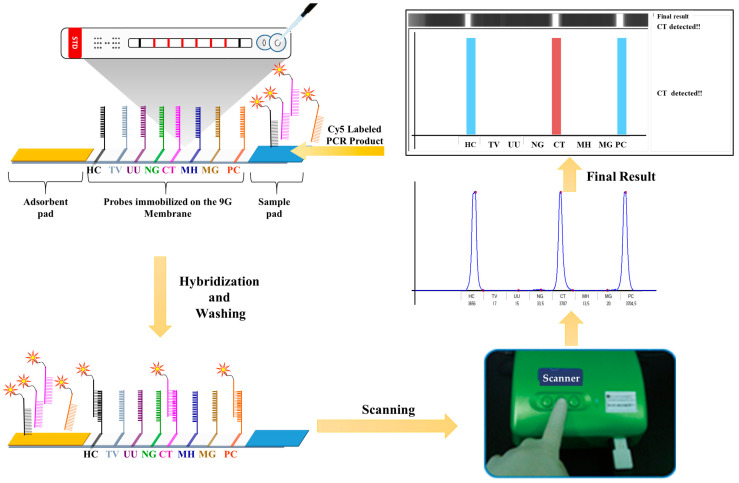
Scheme depicting the workflow and the respective positions of the probes corresponding to CT, NG, TV, UU, MH, MG, PC, and HC on the 6STD Genotyping 9G Membrane, an LFSM.

The 6STD genotyping 9G membranes were obtained by a previously reported method [29,30]. In brief, the oligonucleotide probes P1, P8, P10, P12, P15, and P17 appended with nine consecutive guanines (Table 1) were immobilized by dispensing respective solutions containing 18 pmol/μL of each probe to create eight lines on the AMCA membrane. The dispensed probe solutions were allowed to incubate for 4 h on the AMCA membranes, resulting in the probe immobilization. Following the immobilization of the probes, as illustrated in Scheme 1, the membranes underwent immersion in a blocking solution before being dried to produce the 6STD Genotyping 9G Membranes. In addition to the probes specific to the STD genotypes, probes (Probe6–Probe8) corresponding to the hybridization control (HC), PCR control (PCR), and positive control (PC) were also immobilized on the membranes.

### 2.7. General Procedure of Hybridization, Washing, and Scanning on 6STD Genotyping 9G Membranes

First, 600 μL of Cy5-HC-ssDNA (60 fmol/μL) was mixed with 20 mL of hybridization buffer to produce a hybridization solution. About 240 μL of the hybridization mixture was generated by mixing 220 μL of the hybridization and 20 μL of the Cy5-labeled PCR product specific to one or all of the six STD pathogens (e.g., CT, NG, TV, UU, MH, and MG). About 110 μL of 240 μL of hybridization mixture was applied to the sample loading port on the 6STD genotyping 9G membrane and allowed to hybridize for 20 m at 25 °C. Following hybridization, 240 μL of the washing solution was introduced into the washing port and incubated for 8 m. Subsequently, the 6STD Genotyping 9G Membrane strips were scanned using the BMT Membrane Reader™ to obtain final results. Each experiment was conducted at least three times. The genotyping results can be obtained within 30 m using the 6STD Genotyping 9G Membrane tests.

## 3. Results

### 3.1. Optimization of Primers for Polymerase Chain Reaction (PCR)

**Figure 1:**
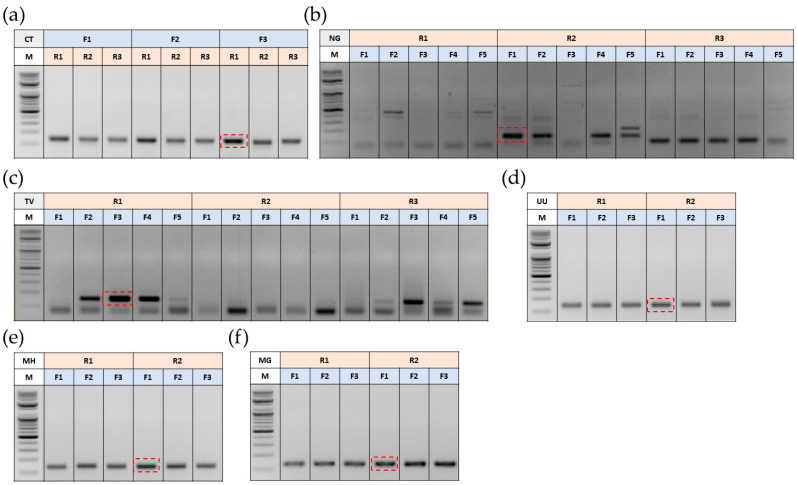
Gel electrophoresis images captured by loading the PCR products obtained using DNA templates and corresponding primer sets of six STD pathogens, including (**a**) *Chlamydia trachomatis* (CT); (**b**) *Neisseria gonorrhoeae* (NG); (**c**) *Trichomonas vaginalis* (TV); (**d**) *Ureaplasma urealyticum* (UU); (**e**) *Mycoplasma hominis* (MH); (**f**) *Mycoplasma genitalium* (MG). M, Marker ladder; F, for word primer; R, reverse primer. Dotted red boxes indicate selected primer sets.

We identified the specific regions on the genomic DNA of each pathogen and procured the synthesized templates from Bioneer Corp., Daejeon, Republic of Korea, as shown in Table S1. We designed several forward and reverse primers (Table S2 in Supplementary Materials) to optimize PCR conditions and select the best primer set for each pathogen. The forward and reverse primer regions for pathogens, UU, MH, and MG, were identical. Hence, the designed primers were the same for these three pathogens. We performed the PCR using the template DNA and the primer sets. The product of these PCR reactions was subjected to gel electrophoresis. The obtained results are presented in Figure 1. As shown in Figure 1a, the primer set comprising F3 and R1 results in high PCR yield and demonstrates high specificity for the DNA template corresponding to CT. Similarly, As shown in Figure 1b–f, the primers R2 and F1 for NG, primers R1 and F3 for TV, primers R2 and F1 for UU, primers R2 and F1 for MH, and primers R2 and F1 for MG demonstrate high specificity and high PCR yield. Thus, we chose these primer sets for further experiments.

### 3.2. Selection of Oligonucleotide Probes for the Development of 6STD Genotyping 9G Membranes

**Figure 2:**
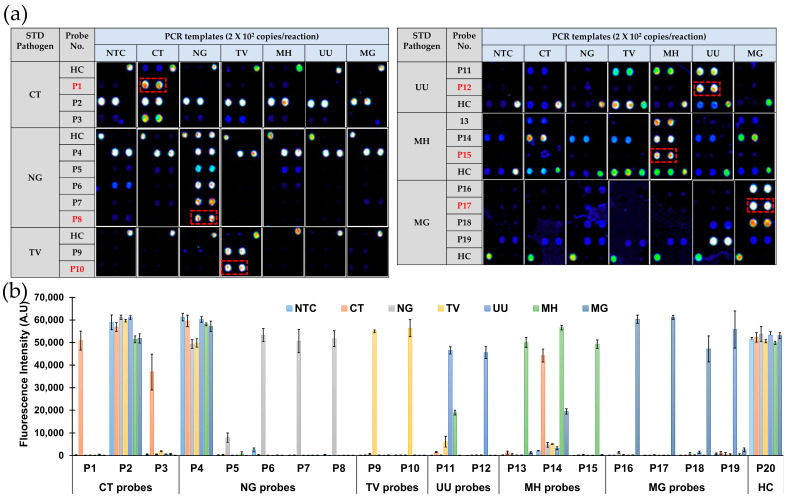
(**a**) Results of hybridization of the PCR products of STD pathogens with the immobilized probes corresponding to each STD pathogen; (**b**) Bar graph representing corresponding signal intensities. The values represent the mean and standard deviation of three replicates. (NTC, Negative control). Text in red font and red boxes indicate optimum probes selected for further study.

Table 1 contains the sequences of the probes we designed based on our previously reported generalized probe selection method for DNA chips. These probes were immobilized on the AMCA slides to generate 9G DNAChips. The PCR products were obtained using the specific templates and selected primer mix corresponding to each STD pathogen. As mentioned earlier, the obtained PCR products were then allowed to hybridize with the immobilized probes on the 9G DNAChips. After hybridization, 9G DNAChips were washed, dried, and scanned using ScanArrayLite (Packard BioChip Technologies, Meriden, CT, USA). The analysis of fluorescence signal intensities was performed using Quant Array software (V3.0). The obtained results are presented in Figure 2.

The signal-to-background ratio (SBR) resulting from the specific and non-specific hybridization of PCR products with immobilized probes plays a crucial role in probe selection. As shown in Figure 2a, when the PCR products of negative control (NTC), CT, NG, TV, UU, MH, and MG were allowed to hybridize with the immobilized probes, they showed specific and non-specific hybridizations. For example, when the immobilized probes corresponding to CT (P1, P2, P3) were allowed to hybridize with the PCR products of NTC, CT, NG, TV, UU, MH, and MG, the probes P2 and P3 demonstrated specific hybridization with the PCR product of CT and non-specific hybridization with the PCR products of other pathogens. However, probe P1 demonstrated specific hybridization with the CT PCR product with a fluorescence intensity value of 56,873, as shown in Figure 2b. The signal intensities for the non-specific interaction of P1 with other PCR products were 110~303, indicating an SBR over 187~517, which is several-fold higher compared to the reported microarray-based DNA–DNA hybridization. Thus, we selected probe P1 for further experiments as it showed high specificity for the PCR product of CT.

Similarly, we chose the probes P8, P10, P12, P15, and P17 for NG, TV, UU, MH, and MG pathogens, respectively, as these probes demonstrated the highly specific hybridization with the PCR products of corresponding pathogens. This highly specific hybridization fluorescence signal intensity (45,526~61,152) and the non-specific hybridization signal intensities (86~312) ensured an SBR of over 500. Hence, we chose probes P1, P8, P10, P12, P15, and P17 for CT, NG, TV, UU, MH, and MG pathogens, respectively, for the development of 6STD Genotyping 9G Membranes, an LFSM assay.

### 3.3. Development of 6STD Genotyping 9G Membranes

The 6STD Genotyping 9G Membranes consist of eight lines corresponding to six specific probes for STD pathogens, including CT, NG, TV, UU, MH, and MG, one line for the positive control (PC), and one line containing the mixture of four hybridization control (HC) probes, as shown in Table 1 and Scheme 1.

As shown in Scheme 1, the workflow for genotyping six STD pathogens is straightforward and does not require a highly trained professional to conduct the assay. In brief, about 110 μL of hybridization mixture containing the Cy5-labeled PCR product obtained from the template DNA or the genomic DNA in the clinical sample using the mixture of selected primers is loaded on the sample loading port of the 6STD Genotyping 9G Membrane for the detection and genotyping of STD pathogens. The immobilized probes are then allowed to hybridize with the PCR product for about 20 min. After hybridization, 100 μL of washing buffer is loaded on the washing port and allowed to stand for 8 min. Upon completion of the washing step, the membranes are scanned on the BMT Membrane Reader™ (Biometrix Technology Inc., Chuncheon, Republic of Korea), providing the fluorescence signal intensities for each line on the membrane. The BMT Membrane Reader™ automatically analyzes the fluorescence signal intensities to give the final results as depicted in Scheme 1.

### 3.4. Evaluation of 6STD Genotyping 9G Membranes

#### 3.4.1. Specificity

**Figure 3:**
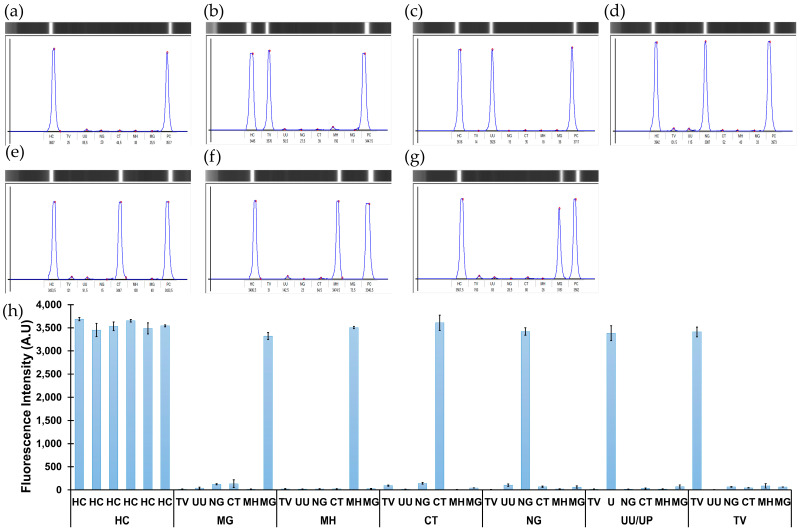
Results of detection and genotyping of (**a**) negative control; (**b**) TV; (**c**) UU; (**d**) NG; (**e**) CT; (**f**) MH and (**g**) MG on 6STD Genotyping 9G Membranes using the PCR products of respective genotypes (genomic DNA template, 1 × 10^5^ copies/test) and the multiplex PCR primer mixture; (**h**) bar graph representing corresponding signal intensities. The values represent the mean and standard deviation of 24 replicates.

To assess the specificity of the 6STD Genotyping 9G Membranes, the immobilized probes were permitted to hybridize with PCR products of six STD pathogens. These PCR products were obtained using the multiplex primer set developed in this study, along with genomic DNA (1 × 10^5^ copies/test) of each pathogen. The experiment was replicated 24 times with high levels of genomic DNA of each pathogen to ensure robustness. The resulting signal intensity graphs captured by the BMT 9G Membrane Reader™ are depicted in Figure 3. As shown in Figure 3a, negative control samples show only specific interactions with the PC (positive control) and HC probes without any non-specific interactions with the probes corresponding to the six STD pathogens. When a TV PCR product was loaded on the 6STD Genotyping 9G Membrane, it showed highly specific hybridization with the immobilized probe P10 on the TV line. The signal intensity for this hybridization was about 3576, and the signal intensities at other lines, including UU, NG, CT, MH, and MG, were only 13~40.5, indicating an SBR of 71~275. These results suggest that the probe P10 corresponding to the TV shows a very high specificity for detecting TV. Similarly, the probes P1, P8, P12, P15, and P17, corresponding to CT, NG, UU, MH, and MG, demonstrated the highly specific detection of respective pathogens with an SBR over 70. Therefore, it can be concluded that the probes immobilized on the 6STD Genotyping 9G Membrane can allow highly specific detection of the six STD pathogens.

#### 3.4.2. Sensitivity (Limit of Detection)

**Figure 4:**
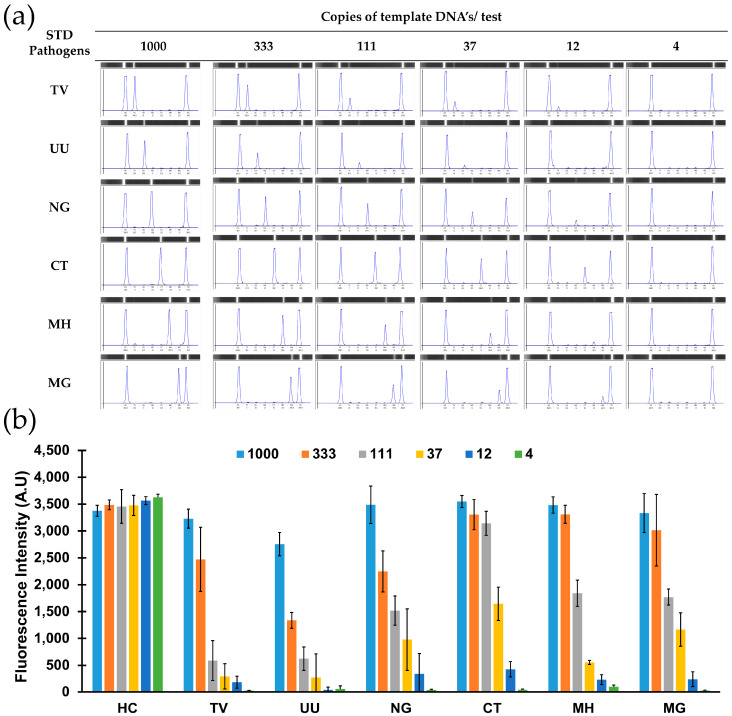
Results of detection and genotyping of (**a**) TV, UU, NG, CT, MH, and MG pathogens on the 6STD Genotyping 9G Membranes using the PCR products obtained from 1000, 333, 111, 37, 12, and 4 copies/test of template DNAs of respective pathogens; (**b**) bar graph representing corresponding signal intensities. The values represent the mean and standard deviation of 24 replicates.

The sensitivity of the 6STD Genotyping 9G Membrane to detect six STD pathogens was determined using the PCR products obtained from the template DNA at concentrations of 1000, 333, 111, 37, 12, and 4 copies per reaction. The obtained PCR products were tested using 6STD Genotyping 9G Membranes. The obtained results are presented in Figure 4. As shown in Figure 4a, all of the probes except the one corresponding to the genotype UU demonstrated signal intensities over the cut-off value of 50 upon hybridization with the PCR products obtained with 12 copies/test of template DNAs. Notably, none of the probes demonstrated significant signal intensities upon hybridization with the PCR products obtained with four copies/tests of template DNAs. We analyzed the data presented in Figure 4b for a 95% LOD by PROBIT at a confidence interval of 95%. We found that the LODs for detecting genotyping of TV, UU, NG, CT, MH, and MG were 63.6, 136.9, 13.4, 33.2, 104.7, and 56.6 copies/test, respectively. These results indicate that the 6STD Genotyping 9G Membranes allow highly sensitive detection and genotyping of six STD genotypes with an excellent limit of detection (LOD) values.

### 3.5. Detection and Genotyping of STD Pathogens in Clinical Samples (Urine) Using 6STD Genotyping 9G Membrane Test

We evaluated the applicability of the 6STD Genotyping 9G Membrane test using 120 clinical samples, as shown in Table 2. All samples were pre-tested by sequencing analysis using the BigDye3 terminator cycle sequencing kit (PE Applied Biosystems, Shelton, CT, USA). In brief, the PCR products obtained following the abovementioned procedure were loaded on the 6STD Genotyping 9G Membrane test to detect STD pathogens in the clinical samples. The results of the clinical study are presented in Table 3.

As shown in Table 3, the 6STD Genotyping 9G Membrane test allows accurate detection and genotyping of each of the STD genotypes, including TV, UU, NG, CT, MH, and MG. The clinical study results presented here were not used to determine the clinical sensitivity and specificity of the 6STD Genotyping 9G Membrane test due to a very small sample size.

## 4. Discussion

STDs pose significant health risks worldwide, manifesting in various symptoms. It is evident from several reports that STDs are curable with antibiotics if the treatment begins early enough [32,33]. However, when they are left untreated, mostly due to late diagnosis, they can cause severe health risks [34,35,36]. The emergence of 1 million new STD cases daily is a severe medical, social, and economic burden to thousands of adults and babies worldwide [37,38,39]. Therefore, with rising incidence rates, there is an urgent need for a robust but highly accurate test that can allow the detection and genotyping of STD pathogens in point-of-care settings [40,41,42].

Sanger sequencing is highly accurate in diagnosing sexually transmitted pathogens but has shortcomings such as long sequencing time, high cost, and complex operation and result interpretation [43]. Sequencing analysis allows the detection of only one pathogen at once. However, most sexually transmitted pathogens can co-infect the hosts [44,45]. Therefore, methods that can detect multiple pathogens simultaneously are important for accurate diagnosis [46,47].

In this context, lateral flow strip membrane assays have emerged as a sensitive and cost-effective alternative requiring less labor, lower reagent costs, and faster multiplex detection [48]. However, the low signal-to-background ratio (SBR) resulting from the specific and non-specific hybridization of the PCR products with the immobilized probes is a known hurdle in developing the LFSM assays [49]. The efficiency of LFSM assays decreases drastically with the decrease in the SBR. Therefore, the design of probes immobilized on the LFSM plays a vital role in the success of the LFSM-based assays. The probes used to develop the 6STD Genotyping 9G membrane test were designed following our previous report on the generalized probe selection method [28]. Remarkably, the probes immobilized on the 6STD Genotyping 9G Membranes exhibited outstanding specificity, resulting in SBR exceeding 70 (Figure 3 and Figure 4). Thus, the 6STD Genotyping 9G Membrane test allows highly specific detection and genotyping of six STD genotypes studied here.

While TaqMan PCR and other PCR techniques offer sensitive methods for detecting and genotyping STD pathogens, they are often associated with time-consuming procedures and false positive results [50]. In contrast, the 6STD Genotyping 9G Membrane tests offer rapid, highly specific genotyping of six different pathogens within 29 m, employing simple hybridization and washing steps suitable for small laboratories without requiring highly trained professionals. The 6STD Genotyping 9G Membrane test demonstrated 100% sensitivity and specificity in detecting and discriminating high-incidence STD genotypes, exhibiting several key advantages: (i) hybridization, washing, and direct scanning at 25 °C, (ii) a high SBR exceeding 70, and (iii) 100% target specificity, as evidenced by identical results with sequencing analysis.

## 5. Conclusions

The 6STD Genotyping 9G membrane test streamlines the detection and genotyping of six STD pathogens, including *Trichomonas vaginalis*, *Ureaplasma urealyticum*, *Neisseria gonorrhoeae*, *Chlamydia trachomatis*, *Mycoplasma hominis*, and *Mycoplasma genitalium* in clinical samples through a straightforward experimental procedure. The 20 m hybridization step, 8 m washing step, and less than a 1 m scanning step allowed us to obtain the final detection and genotyping results in less than 30 m after PCR at 25 °C using the 6STD Genotyping 9G Membrane test. Scanning the membrane requires no drying steps or special handling, making this test user-friendly for precise STD genotyping. The accuracy of the 6STD Genotyping 9G membrane test in STD genotyping was confirmed by its 100% concordance with sequencing analysis results. This test holds clinical significance by accurately detecting the six STD pathogens in the clinical samples. Therefore, the 6STD Genotyping 9G Membrane test emerges as a promising diagnostic tool for precise STD genotyping, facilitating informed decision-making in clinical practice.

## Figures and Tables

**Table 1:** Probe sequences corresponding to the 6 STD pathogens hybridization control (HC).

STD Pathogen	Probe Name	Probe Sequence (5′ to 3′)	Tm (°C)	GC %
CT	**P1**	**GGGGGGGGG AAATACAAA TGAGGCTGATGACTAGGATG**	**61.0**	**50.0**
	P2	GGGGGGGGG AAATACAAA CATGGGAGTTGGTTTTACCT	60.8	45.0
	P3	GGGGGGGGG AAATACAAA CCAAGGTGAGGCTGATGACT	64.7	55.0
NG	P4	GGGGGGGGG AAATACAAA AGTGGGGGATACCAGAAGTA	60.4	50.0
	P5	GGGGGGGGG AAATACAAA ACCGCAAGGAGTCCGCTTAC	67.8	60.0
	P6	GGGGGGGGG AAATACAAA ACCACGGTATGCTTCATGAC	62.9	50.0
	P7	GGGGGGGGG AAATACAAA TCCGCTGACCACGGTATGCT	70.4	60.0
	**P8**	**GGGGGGGGG AAATACAAA CGCTGACCACGGTATGCTTC**	**68.2**	**60.0**
TV	P9	GGGGGGGGG AAATACAAA TGGATGACTCGGTGAAATCA	64.7	45.0
	**P10**	**GGGGGGGGG AAATACAAA AATCACGTTATCTAGAGGAAGG**	58.4	40.9
UU	P11	GGGGGGGGG AAATACAAA GCTAACCTTTTGGAGGCATG	63.8	50.0
	**P12**	**GGGGGGGGG AAATACAAA ATGCGTCTAGGGTAGGATCG**	**63.3**	**55.0**
MH	P13	GGGGGGGGG AAATACAAA TTGCTAACCTCGGAGGCGAC	68.9	60.0
	P14	GGGGGGGGG AAATACAAA CTAAGGTAGGACTGGTGACT	55.4	50.0
	**P15**	**GGGGGGGGG AAATACAAA GACCGCCTAAGTTAGGACTG**	**60.8**	**55**
MG	P16	GGGGGGGGG AAATACAAA GAAGTGCATGTCAAGGATAGC	61.7	47.6
	**P17**	**GGGGGGGGG AAATACAAA CCTTTATTGGAAGTGCATGTC**	**61.4**	**42.9**
	P18	GGGGGGGGG AAATACAAA CCTTTATTGGAAGTGCTTGTC	60.6	42.9
	P19	GGGGGGGGG AAATACAAA CTATCCTTTATTGGAAGTGC	56.0	40.0
HC	P20 *	**GGGGGGGGG AAATACAAA AAGGATAAGGAAGAAGCCTG**	**59.4**	**45.0**
		**GGGGGGGGG AAATACAAA GTTCTAGTTTTAATAACTAACAC**	**48.8**	**26.1**
		**GGGGGGGGG AAATACAAA CTAGAGAAAGAAGGGGCTTT**	**58.7**	**45.0**
		**GGGGGGGGG AAATACAAA CCTACGAGAACGTGGGGATG**	**67.0**	**60.0**

GGGGGGGGG, nine consecutive guanines for immobilizing the probes on the AMCA slides; AAATACAAA—vertical spacer group; * is a hybridization control (HC) probe containing a 1:1:1:1 mixture of the four probes. The final probes selected for the 6STD Genotyping Membrane test fabrication are in boldface; Tm, melting temperature; CG (%), percentage of nucleotides cytosine and guanine in the sequence; Bold text, Probe sequences given in bold text were finalized for the generation of 6STD Genotyping 9G Membranes.

**Table 2:** The applicability of the 6STD Genotyping 9G Membrane test was evaluated using 120 urine samples.

STD Pathogens	Positive Samples	Negative Samples
TV	10	60
UU	10	
NG	10	
CT	10	
MH	10	
MG	10	

**Table 3:** Comparison of the results of sequencing analysis and the results of the 6STD Genotyping 9G Membrane test for the detection and genotyping of STD pathogens in 120 clinical samples.

STD Pathogens	Total Samples	Results of 6STD Genotyping 9G Membrane	Results of Sequencing Analysis
TV	10	10	10
UU	10	10	10
NG	10	10	10
CT	10	10	10
MH	10	10	10
MG	10	10	10
Negative	60	60	60

## Data Availability

The data presented in this study are available upon request from the corresponding author.

## Supplementary Materials

The following supporting information can be downloaded at: https://www.mdpi.com/article/10.3390/bios14050260/s1, Table S1: DNA templates used for the design and development of probes for the detection of STD pathogens, including *Chlamydia trachomatis* (CT), *Neisseria gonorrhoeae* (NG), *Trichomonas vaginalis* (TV), *Ureaplasma urealyticum* (UU), *Mycoplasma hominis* (MH), and *Mycoplasma genitalium* (MG).; Table S2: List of primer candidates used to develop 6 STD Genotyping 9G Test.
