# Supplementary material for: Development of a DNA-Based Lateral Flow Strip Membrane Assay for Rapid Screening and Genotyping of Six High-Incidence STD Pathogens
Source: Biosensors (Basel). 2024 May 20;14(5):260. doi: 10.3390/bios14050260 (PMC11118844; doi:10.3390/bios14050260)
Supplement: Supplementary file 1 [file biosensors-14-00260-s001.zip › biosensors-2985546-supplementary.pdf]

biosensors

MDPI

Supplementary Materials

Development of a DNA-Based Lateral Flow Strip Membrane Assay for Rapid Screening and Genotyping of Six High-Incidence STD Pathogens

Gunho Choi [1], Keum-Soo Song [1], Satish Balasaheb Nimse [2,*] and Taisun Kim [2,*]

[1] Biometrix Technology, Inc., 2-2 Bio Venture Plaza 56, Chuncheon 24232, Republic of Korea; ghchoi@bmtchip.com (G.C.); hanlimsk@empas.com (K.-S.S.)
[2] Institute of Applied Chemistry and Department of Chemistry, Hallym University, Chuncheon 24252, Republic of Korea
* Correspondence: satish_nimse@hallym.ac.kr (S.B.N.); tskim@hallym.ac.kr (T.K.)

Table S1. DNA templates used for the design and development of probes for the detection of STD pathogens, including *Chlamydia trachomatis* (CT), *Neisseria gonorrhoeae* (NG), *Trichomonas vaginalis* (TV), *Ureaplasma urealyticum* (UU), *Mycoplasma hominis* (MH), and *Mycoplasma genitalium* (MG).

STD Pathogen	Blast Reference for 16S Ribosomal RNA Partial Gene	Sequence
***Chlamydia trachomatis* (CT)**	CP017744.1	CAATGGCCAGTACAGAAGGTAGCAAGATC<br>GTGAGATGGAGCAAATCCTCAAAGCTGGCC<br>CCAGTTCGGATTGTAGTCTGCAACTCGACT<br>ACATGAAGTCGGAATTGCTAGTAATGGCGT<br>GTCAGCCATAACGCCGTGAATACGTTCCCG<br>GGCCTTGTACACACCGCCCGTCACATCATG<br>GGAGTTGGTTTTACCTTAAGTCGTTGACTCA<br>ACCCGCAAGGGAGAGAGGCGCCCAAGGTG<br>AGGCTGATGACTAGGATGAAGTCGTAACAA<br>GGTAGCCCTACCGGAAGGTGGGGCTGGATC<br>ACCTCCTTTTAAGGATAAGGAAGAAGCCTG<br>AGAAGGTTTCTGACTAGGTTGGGCAAGCAT<br>TTATATGTAAGAGCAAGCATTCTATTTCATT<br>TGTGTTGTTA
***Neisseria gonorrhoeae* (NG)**	CP032429.2	ATGGTCGGTACAGAGGGTAGCCAAGCCGC<br>GAGGCGGAGCCAATCTCACAAAACCGATC<br>GTAGTCCGGATTGCACTCTGCAACTCGAGT<br>GCATGAAGTCGGAATCGCTAGTAATCGCAG<br>GTCAGCATACTGCGGTGAATACGTTCCCGG<br>GTCTTGTACACACCGCCCGTCACACCATGG<br>GAGTGGGGGATACCAGAAGTAGGTAGGGT<br>AACCGCAAGGAGTCCGCTTACCACGGTATG<br>CTTCATGACTGGGGTGAAGTCGTAACAAGG<br>TAGCCGTAGGGGAACCTGCGGCTGGATCAC<br>CTCCTTTCTAGAGAAAGAAGGGGCTTTAGG<br>CATTCACACTTATCGGTAAACTGAAAAGAT<br>GCGGAAGAAGCTTGAGTGAAGGCAAGGTT<br>CGCTTAAGAAGGGA

***Trichomonas vaginalis* (TV)**	JX943583.1	CAATGTTAGGATCAATAGGACTGCGAGCCT<br>GAGAGGGTGCGCTACTCTTATAATCCCTAA<br>CGTAGTTGGGATTGACGTTTGTAATCAGCGT<br>CATGAACCAGGAATCCCTTGTAAATGTGTG<br>TCAACAACGCACGTTGAATACGTCCCTGCC<br>CTTTGTACACACCGCCCGTCGCTCCTACCGA<br>TTGGATGACTCGGTGAAATCACCGGATGCT<br>TACGAGCAGAAAGTGATTAAATCACGTTAT<br>CTAGAGGAAGGAGAAGTCGTAACAAGGTA<br>ACGGTAGGTGAACCTGCCGTTGGATCAGTT<br>CTAGTTTTAATAACTAACACCAACTTCTTTT<br>ATTAAACAAAAACCAATACAAAATTAAAA<br>ACTAACTTCATCAAAAACCAAGTCTCTAAG<br>CAATGGATG
***Ureaplasma urealyticum* (UU)**	CP039963.1	AAAAGTTAGTCTCAGTTCGGATAGAGGGCT<br>GCAATTCGCCCTCTTGAAGTTGGAATCACT<br>AGTAATCGCGAATCAGACATGTCGCGGTGA<br>ATACGTTCTCGGGTCTTGTACACACCGCCCG<br>TCAAACTATGGGAGCTGGTAATATCTAAAA<br>CCGCAAAGCTAACCTTTTGGAGGCATGCGT<br>CTAGGGTAGGATCGGTGACTGGAGTTAAGT<br>CGTAACAAGGTATCCCTACGAGAACGTGGG<br>GATGGATCACCTCCTTTCTTCGGAGTAAATT<br>TTTAATTTACGTACTAATAAGTGTACATTTT<br>TATAAAAATCCATGTGAATATTAGCCACTTT<br>TTTAAAAAATATTTCAAAAGTTCATATGGTC<br>GGATTCTATTTAGTTTTGAGAGTTTATTCTCT<br>CCC
***Mycoplasma hominis* (MH)**	FP236530.1	ACAATGGTCGGTACAAAGAGAAGCAATAT<br>GGCGACATGGAGCAAATCTCAAAAAGCCG<br>ATCTCAGTTCGGATTGGAGTCTGCAATTCGA<br>CTCCATGAAGTCGGAATCGCTAGTAATCGC<br>AGATCAGCTATGCTGCGGTGAATACGTTCT<br>CGGGTCTTGTACACACCGCCCGTCACACCA<br>TGGGAGCTGGTAATACCCAAAGTCGGTTTG<br>CTAACCTCGGAGGCGACCGCCTAAGGTAGG<br>ACTGGTGACTGGGGTGAAGTCGTAACAAGG<br>TATCCCTACGAGAACGTGGGGATGGATCAC<br>CTCCTTTCTACGGAGTACAACCTATGTTATG<br>GAAAAAATATTTGTATCCAGTTTTGAGAGA<br>TTTATCTCTCGGTTCTTTGAAAACTGAATAT<br>CGACATTGA
***Mycoplasma genitalium* (MG)**	CP003773.1	CCAACTTGTAAAAGTGAGCAAATCTGAAAA<br>GTTGGTCTCAGTTCGGATTGAGGGCTGCAAT<br>TCGTCCTCATGAAGCTGGAATCACTAGTAA<br>TCGCGAATCAGCTATGTCGCGGTGAATACG<br>TTCTCGGGTCTTGTACACACCGCCCGTCAAA<br>CTATGAAAGCTGGTAATATTTAAAAACGTG<br>TTGCTAACCTTTATTGGAAGTGCATGTCAAG<br>GATAGCACCGGTGATTGGAGTTAAGTCGTA<br>ACAAGGTACCCCTACGAGAACGTGGGGT

	GGATCACCTCCTTTCAAATGGAGTTTTTATTTTTTTATTTATCTTAAACACCCATTAATTTTTTCGGTGTTAAAACCCAAATCAATGTTTGGTCTCACAACTAACACATTTGGTCAGTTTGTATCCAG

Table S2. List of primer candidates used to develop 6 STD Genotyping 9G Test.

STD Pathogen	Primer Names	Sequence (5′ - 3′)	Tm (°C)	CG (%)
Chlamydia trachomatis (CT)	CT-F1	CATCATGGGAGTTGGTTTTACCTT	66.0	41.7
	CT-F2	GTTGGTTTTACCTTAAGACGTTGACTC	64.9	40.7
	CT-F3	GTTGACTCAACCAGCAAGGGAG	67.4	54.5
	CT-R1	CTCAGGCTTCTTCCTTATCCTT	62.0	45.5
	CT-R2	TAGGGCTACCTTGTTACGACTT	61.0	45.5
	CT-R3	GAAGCCTGAGAAGGTTTCTGAC	63.5	50.0
Neisseria gonorrhoeae (NG)	NG-F1	GGTAGGGTAATCGCAAGGAGTCC	67.8	56.5
	NG-F2	GGAGTGGGGGATACCAGAAGTAG	66.0	56.5
	NG-F3	GGGGATACCAGAAGTAAGTAGGGTA	63.5	48
	NG-F4	GTAACCTCAAGGAGTCCGCTTAC	64.3	52.2
	NG-F5	GGGATACCAGCAGTAAGTAGGGTA	63.4	50.0
	NG-R1	TACGGCTACCTTGTTACGACTT	61.9	45.5
	NG-R2	CCTAAAGCCCCTTCTTTCTCTAG	62.7	47.8
	NG-R3	ACCGATAAGTGTGAATGCCTAAAG	64.3	41.7
Trichomonas vaginalis (TV)	TV_F1	ATTGGATGACTCGGTGAAATCA	65.7	40.9
	TV_F2	CTCGGTGAAATCACTGGATGCTTA	68.3	45.8
	TV_F3	GGTGAAATCACTGGATGCTTACGA	68.2	45.8
	TV_F4	TGACTCGATGAAATCACCGGATG	70.4	47.8
	TV_F5	GTGAAATCACTGGATGCTTACGA	65.3	43.5
	TV-R1	TGGTGTTAGTTATTAAAACTAGAACTG	58.2	29.6
	TV-R2	TACCGTTACCTTGTTACGACTT	59.6	40.9
	TV-R3	GGTTTTTGTTTAATAAAAGAAGTTGGTG	63.7	28.6
Ureaplasma urealyticum (UU), *Mycoplasma hominis* (MH),	UU-MH-MG_F1	CGTCACACTATGAGAGCTGGTAAT	63.6	45.8
	UU-MH-MG_F2	CGTCACACTATGAGAGCTGGTAATA	63.5	44
	UU-MH-MG_F3	GTCACACTATGAGAGCTGGTAATA	59.4	41.7

***Mycoplasma genitalium* (MG)**	UU-MH-MG-R1	CCCACGTTCTCGTAGGGATA	64.1	55.0
	UU-MH-MG-R2	CATCCCCACGTTCTCGTAGG	67.0	60.0

F, forward primer; R, reverse primer; Tm, melting temperature; Tm, melting temperature; CG (%), percentage of nucleotides cytosine and guanine in the sequence; Red color sequences, these forward and reverse primer sequences were found to be optimum for PCR of the respective STD pathogen.
